# BTF3 confers oncogenic activity in prostate cancer through transcriptional upregulation of Replication Factor C

**DOI:** 10.1038/s41419-020-03348-2

**Published:** 2021-01-05

**Authors:** Yuan Zhang, Xiang Gao, Jingyan Yi, Xiaolin Sang, Zhihong Dai, Zhiwei Tao, Min Wang, Lanlin Shen, Yaxun Jia, Daqing Xie, Hailing Cheng, Zhiyu Liu, Pixu Liu

**Affiliations:** 1 Cancer Institute, Department of Urology, The Second Hospital of Dalian Medical University; Institute of Cancer Stem Cell, Dalian Medical University, Dalian, Liaoning China; 2 Department of Urology, The Second Hospital of Dalian Medical University, Dalian, Liaoning China; 3 Institute of Cancer Stem Cell, Dalian Medical University, Dalian, Liaoning China

**Keywords:** Oncogenes, Tumour biomarkers, Prostate cancer, Mechanisms of disease

## Abstract

High levels of Basic Transcription Factor 3 (BTF3) have been associated with prostate cancer. However, the mechanisms underlying the role of BTF3 as an oncogenic transcription factor in prostate tumorigenesis have not been explored. Herein, we report that BTF3 confers oncogenic activity in prostate cancer cells. Mechanistically, while both BTF3 splicing isoforms (BTF3a and BTF3b) promote cell growth, BTF3b, but not BTF3a, regulates the transcriptional expression of the genes encoding the subunits of Replication Factor C (RFC) family that is involved in DNA replication and damage repair processes. BTF3 knockdown results in decreased expression of RFC genes, and consequently attenuated DNA replication, deficient DNA damage repair, and increased G2/M arrest. Furthermore, knockdown of the RFC3 subunit diminishes the growth advantage and DNA damage repair capability conferred by ectopic overexpression of BTF3b. Importantly, we show that enforced BTF3 overexpression in prostate cancer cells induces substantial accumulation of cisplatin-DNA adducts and render the cells more sensitive to cisplatin treatment both in vitro and in vivo. These findings provide novel insights into the role of BTF3 as an oncogenic transcription factor in prostate cancer and suggest that BTF3 expression levels may serve as a potential biomarker to predict cisplatin treatment response.

## Introduction

Prostate cancer is one of the most common causes of cancer death in men worldwide. Metastatic prostate cancer may initially respond to androgen deprivation, but inevitably develop resistance to therapeutics and subsequently progress to castration-resistant prostate cancer (CRPC)^1,2^. Platinum-based compounds are highly potent anti-tumor drugs in a variety of cancer types, acting through the formation of covalent cisplatin-DNA adducts^3–5^. These compounds exhibit only moderate to good anti-tumor activity in molecularly unselected patients with advanced prostate cancer^6^. Interestingly, however, complete responders have been reported in the clinical trials with cisplatin-based therapies^7–10^. There remain important unmet medical needs to identify biomarkers to predict treatment response to platinum-based chemotherapy and improve the prognosis of prostate cancer patients.

Cancer-causing transcription factors are frequently deregulated in human malignant tumors, many of which have been demonstrated as drivers of tumor growth^11–13^. Transcriptional dysregulation induced by aberrant transcription factors plays a crucial role in tumor progression and therapeutic responses^14^. Hence understanding how transcription factors promote tumorigenesis through dysregulation of their target gene expression may help identify predictive markers of drug sensitivity in prostate cancer and improve the prognosis of patients^11,13,15^.

Basic transcription factor 3 (BTF3), also known as the beta subunit of the nascent polypeptide-associated complex, was originally reported to be a member of general transcription machinery and form a stable complex with the RNA polymerase II to initiate transcription^16,17^. BTF3 exists in two splicing isoforms, BTF3a and BTF3b. BTF3b lacks the N-terminal 44 amino acids of BTF3a^17,18^. It was previously reported that while both isoforms are able to bind to RNA polymerase II, BTF3a but not BTF3b is transcriptionally active in in vitro assays^18^. Nevertheless, both BTF3a and BTF3b have been shown to interact with human estrogen receptor α (ERα) and regulate ERα-mediated transcription in luminal breast cancer cells^19,20^. In addition to its transcriptional activity, BTF3 may have some other functions. For example, a recent study reported that BTF3 protein positively regulates CBF target gene expression through promoting the stability of CBFs during plant cold stress^21^.

In human cancer, BTF3 overexpression has been found in a wide range of cancer types including glioma^22^, hepatocarcinoma^23^, pancreatic ductal adenocarcinoma^24^, gastric cancer^25,26^, nasopharyngeal carcinoma^27^, and prostate cancer^28,29^. More recently, BTF3 has been shown to sustain prostate cancer stemness via interaction and stabilization of BMI1^29^. Given the transcriptional activity of BTF3 reported in other cancer types^19,24^, whether BTF3 may contribute to tumorigenicity in prostate cancer through its transcriptional activity remains unknown. In the current study, we investigated the biological role of BTF3 as an oncogenic transcription factor and its potential as a predictive biomarker for the sensitivity of chemotherapy in prostate cancer.

## Results

### BTF3 plays an oncogenic role in prostate cancer in vitro and in vivo

**Fig. 1:**
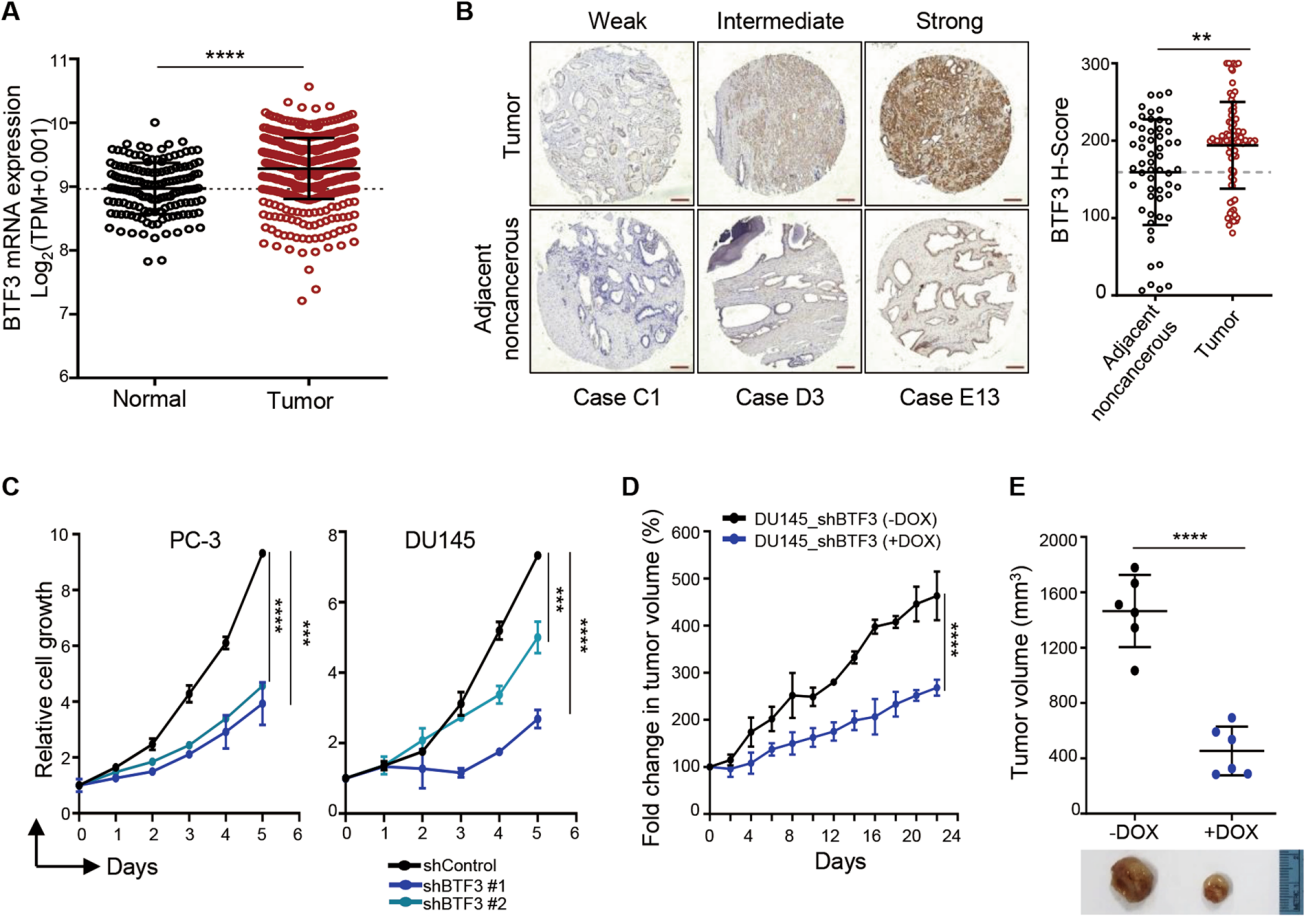
BTF3 plays an oncogenic role in prostate cancer in vitro and in vivo. **a** BTF3 expression levels in prostate cancer and normal tissue. Transcriptome data of prostate tumors from TCGA and normal tissues from TCGA and GTEx samples were obtained from the UCSC Xena (https://xena.ucsc.edu). The gene expression is reported as log_2_(Transcripts per million (TPM) + 0.001). Each dot represents an individual sample (*n* = 496 for prostate tumor; *n* = 149 for normal tissue. The black lines in each group indicate the mean ± S.D. ****p < 0.0001 (Mann–Whitney test). **b** Immunohistochemistry staining of tissue microarray from prostate tumors and adjacent noncancerous tissues using anti-BTF3 antibody. Representative images of BTF3 stained tumor and their corresponding noncancerous tissue sections are shown in weak, Intermediate, and strong staining, respectively. The scatter plot graph showing a statistical analysis of BTF3 expression (H score) in tumor and adjacent noncancerous tissues. Data are shown as mean ± S.D.; *n* = 82 for tumors and *n* = 55 for adjacent noncancerous tissues. Scale bar, 25 μm. **p < 0.01 (Mann-Whitney test). **c** Relative cell growth of PC-3 and DU145 prostate cancer cells with or without BTF3-knockdown was measured by crystal violet assay. Data are shown as mean ± S.D. for three independent experiments. ***p < 0.001, ****p < 0.0001 (Student’s *t*-test). **d**, **e** DU145-Tet-On-shBTF3 xenografted mice were treated with or without doxycycline. The graph shows the fold change in tumor volume, with respect to the initial treatment at day 0 (**d**). The tumor volume and representative images of dissected tumors at the endpoint of treatment are shown (**e**). Dox, Doxycycline (2 mg/ml in drinking wat**e**r). The data are shown as mean ± S.E.M (*n* = 6 for each group). ****p < 0.0001 (Student’s *t*-test).

Analysis of publicly available clinical data revealed significantly increased BTF3 mRNA expression in prostate cancer compared to normal control (Fig. 1a, TCGA prostate cancer). We also conducted immunohistochemical staining of BTF3 protein in a panel of primary human prostate tumor samples with adjacent non-cancerous tissues. BTF3 was largely present in epithelial cancer cells and the abundance of BTF3 protein was significantly elevated in prostate cancer tissues relative to the matched para-tumor tissues (Fig. 1b). These data prompted us to investigate the oncogenic potential of BTF3 in prostate cancer. Indeed, silencing of BTF3 via shRNA resulted in significantly attenuated growth of PC-3 and DU145 prostate cancer cells cultured in both 2D and 3D conditions, enhanced apoptotic cell death (Fig. 1c and Supplementary Fig. S1a–e), as well as mitigated migration and invasion potential (Supplementary Fig. S1f–g). Together, these in vitro data support an oncogenic role of BTF3 in prostate cancer^29^.

We next sought to examine if BTF3 may confer oncogenic activity in vivo. For this, we established a DU145 cell line that allows doxycycline-inducible expression of BTF3-specific shRNA. We confirmed that the doxycycline treatment resulted in remarkable downregulation of BTF3 expression at mRNA and protein levels (Supplementary Fig. S2a, b). Inducible knockdown of BTF3 expression led to attenuated growth of DU145 cells cultured in both 2D monolayer and 3D Matrigel conditions (Supplementary Fig. S2c, d), similar to the effects of straight shRNA-mediated silencing of BTF3 (Fig. 1c and Supplementary Fig. S1c, d). Importantly, doxycycline-induced BTF3 knockdown significantly attenuated the growth of DU145-derived xenografts (Fig. 1d, e), validating the oncogenic role of BTF3 in prostate tumor development.

### BTF3 is involved in the regulation of DNA replication and DNA damage repair in prostate cancer cells

**Fig. 2:**
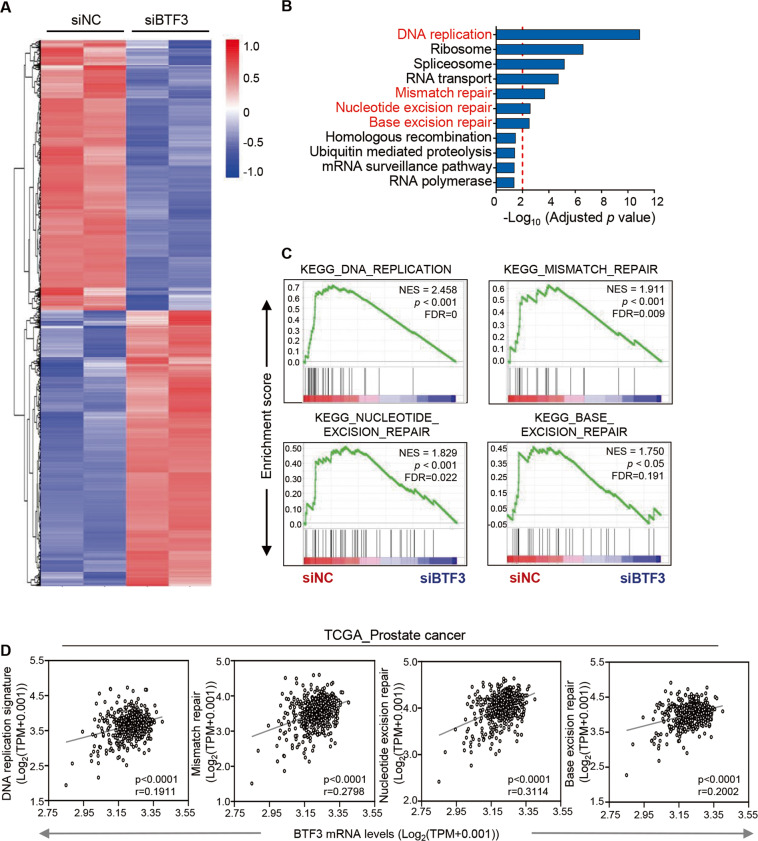
BTF3 expression associates with DNA replication and DNA damage repair processes in prostate cancer cells. **a** Heat map expression plot of up-regulated and down-regulated genes in siBTF3 #1 transfected PC-3 cells versus control cells (siNC) as revealed by RNA sequencing. The gene expression was calculated according to the FPKM value. Genes with adjusted *p*-values less than 0.05 are shown. **b** KEGG pathway enrichment analysis of down-regulated gene sets upon BTF3 knockdown in PC-3 cells. Pathways with adjusted *p*-value less than 0.05 are shown. The threshold of adjusted *p*-value = 0.01 is shown as red dotted line. **c** GSEA analysis of the DNA damage repair gene signatures as indicated in siBTF3 transfected PC-3 cells versus control cells. Normalized enrichment score (NES), *p*-value and False Discovery Rate (FDR) q value of the correlation are shown. **d** BTF3 gene expression was plotted against the DNA replication and DNA damage repair signatures as indicated for TCGA prostate cancer datasets (*n* = 496). Gene expression levels are reported as log_2_ (TPM + 0.001). Each dot represents an individual sample of human prostate tumors. The linear regression Spearman’s correlation coefficient (r) and its *p*-values are indicated.

To gain insights into the molecular mechanism underlying the oncogenic role of BTF3 in prostate cancer, we performed RNA sequencing analysis to assess the effect of BTF3 silencing by siRNA on specific transcriptional changes (Supplementary Fig. S3a–d and Fig. 2a). Gene set enrichment and pathway analyses revealed a significant association of BTF3 expression with gene expression related to DNA replication, mismatch repair, nucleotide excision repair and base excision repair (Fig. 2b, c). In line with these findings, analyses of TCGA_prostate cancer cohort also yielded a modest but significant correlation between BTF3 and gene signatures involved in DNA replication and DNA damage repair processes (Fig. 2d). These findings, for the first time, point to a novel role of BTF3 in the regulation of DNA replication and DNA damage repair.

**Fig. 3:**
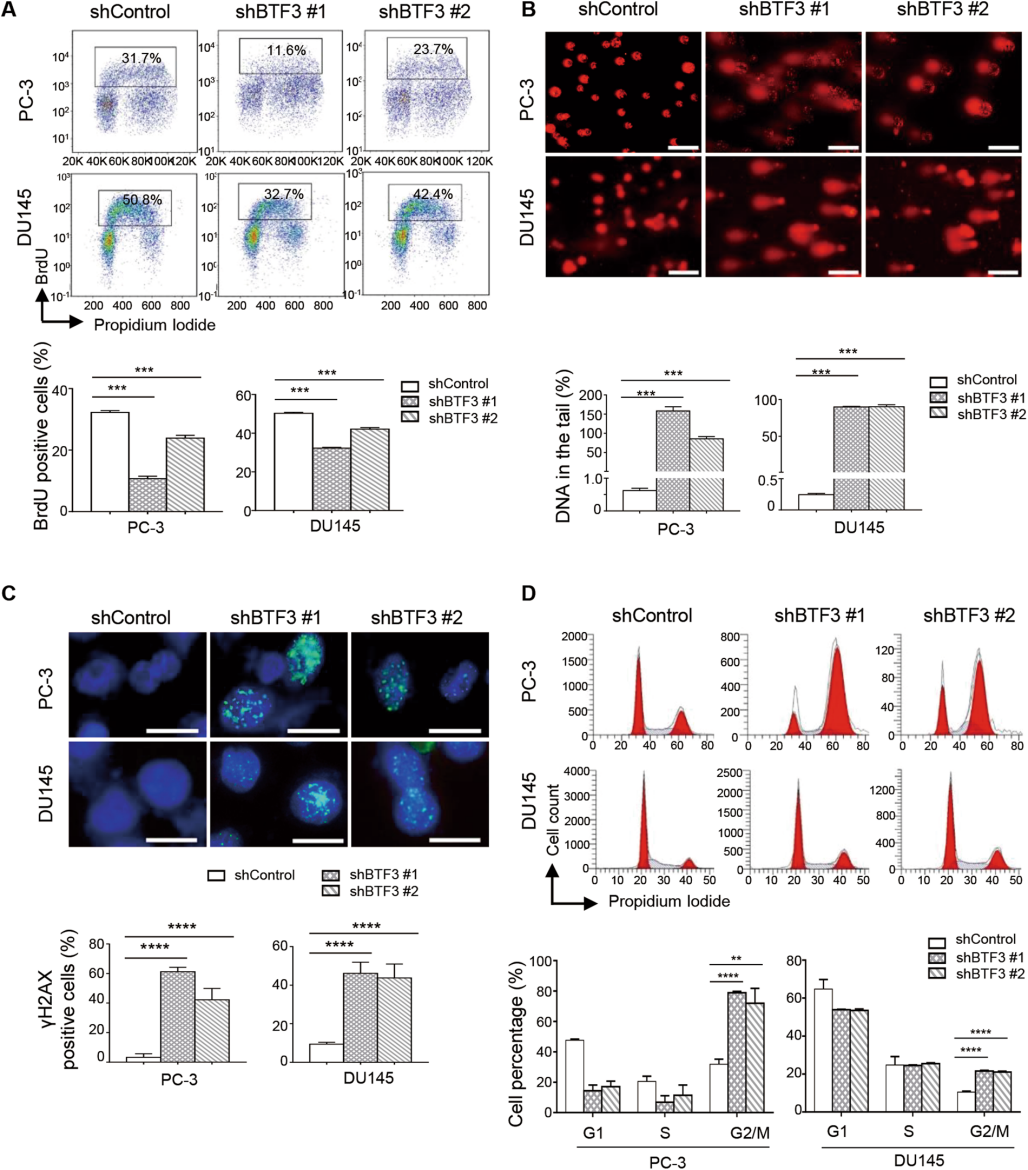
BTF3 knockdown resulted in reduced DNA replication, induced DNA damage and G2/M arrest in prostate cancer cells. **a** Flow cytometric analysis of BrdU incorporation was used to determine the S-phase entry in PC-3 and DU145 cells with or without BTF3-knockdown. Scatter plots of newly synthesized DNA (BrdU positive) versus total DNA (PI positive) are shown. The quantification of BrdU positive cells is shown as mean ± S.D. ***p < 0.001 (Student’s *t*-test). **b** Comet assay was performed to evaluate DNA damage in cells as in (**a**). Quantification of DNA in the tail from three independent experiments is shown as mean ± S.D. Scale bar, 100 μm. ***p < 0.001 (Student’s *t*-test). **c** Immunofluorescence staining of γH2AX (green) and DAPI (blue) in cells as in (**a**). Cells containing more than five foci were scored as positive. Mean ± S.D. for three independent experiments is shown. Scale bar, 20 μm. ****p < 0.0001 (Student’s *t*-test). **d** Flow cytometric analysis of the cell cycle for PC-3 and DU145 cells with or without BTF3-knockdown. Quantification of cells in each phase is shown as mean ± S.D. for three independent experiments. **p < 0.01, ****p < 0.0001 (Student’s *t* -test).

To explore the link of BTF3 to DNA replication, we conducted flow cytometry analysis of 5-bromodeoxoyuridin (BrdU) incorporation. shRNA-mediated stable knockdown of BTF3 in PC-3 and DU145 cells resulted in significantly reduced percentage of BrdU positive cells (Fig. 3a), a marker of replicative DNA synthesis in S-phase, consistent with a role for BTF3 in DNA replication in prostate cancer cells. We next assessed the potential role of BTF3 in DNA damage response. BTF3 knockdown resulted in robust amounts of DNA in the comet tails of PC-3 and DU145 cells (Fig. 3b), an indicator of DNA strand breaks caused by BTF3 silencing. Concordantly, BTF3 knockdown in PC-3 and DU145 cells induced a substantial accumulation of γH2AX nuclear foci (Fig. 3c), a surrogate marker for DNA double strand break damage^30^. These data support an important role of BTF3 in DNA damage repair. Accumulating evidence indicates that cell cycle arrest in G2/M phase often occurs in response to DNA damage^31^. In line with this, flow cytometry analysis revealed an increased percentage of cells in the G2/M phase of the cell cycle in both BTF3-knockdown PC-3 and DU145 cells (Fig. 3d). Taken together, these results suggest an important role of BTF3 in the regulation of DNA replication and DNA damage repair in prostate cancer cells.

### BTF3b exerts its oncogenic effects through transcriptional regulation of RFCs in prostate cancer

**Fig. 4:**
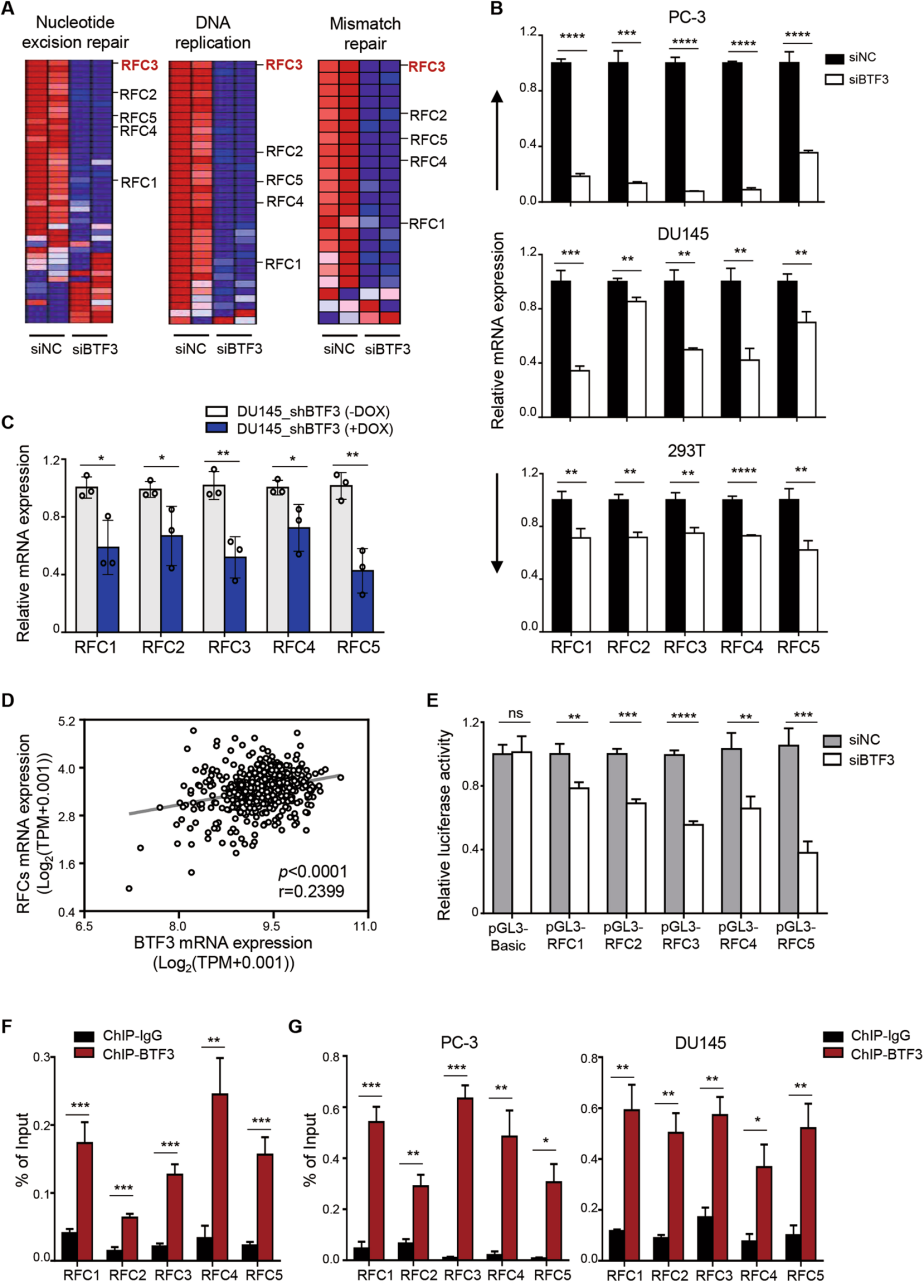
BTF3 transcriptionally upregulates the expression of RFC family genes in prostate cancer cells. **a** Heat map plot of differentially expressed genes involved in DNA replication, nucleotide excision repair and mismatch repair in siBTF3 transfected PC-3 cells or control cells. **b** Quantitative reverse transcription-PCR (qRT-PCR) analysis of the *RFCs* mRNA levels was conducted in cells as indicated. Data are shown as mean ± SD for three independent experiments (**p < 0.01, ***p < 0.001, ****p < 0.0001 by Student’s *t*-test). **c** qRT-PCR analysis of the *RFCs* mRNA levels was conducted in DU145-Tet-On-shBTF3 xenografted tumors treated with or without Dox for 22 days. *ACTB* was used as an endogenous control. Dox, Doxycycline, 2 mg/ml in drinking water. Data are shown as mean ± S.D. *p < 0.05, **p < 0.01 (Student’s *t*-test). **d** The gene expression levels of BTF3 and RFC family genes were examined in the TCGA prostate cancer datasets (*n* = 496). The gene expression levels are reported as log_2_(TPM + 0.001) and plotted as BTF3 gene expression over RFC family genes expression. Each dot represents an individual sample of human prostate tumor. Spearman’s correlation coefficient (r) and *p*-values were determined as indicated. **e** The RFC promoter luciferase reporter assay was perform**e**d in siNC or siBTF3 transfected HEK293T cells. pGL3-Basic, control vector; pGL3-RFC1/2/3/4/5, vectors carrying promoter sequence from RFC1, RFC2, RFC3, RFC4 and RFC5, respectively. Data are shown as mean ± S.D. n.s, not significant, **p < 0.01, ***p < 0.001, ****p < 0.0001 (Student’s *t*-test). **f**, **g** Chromatin immunoprecipitation (ChIP) assay was performed by BTF3 chromatin immunoprecipitation followed by quantitative PCR for the RFC promoters in HEK 293T cells (**f**), PC-3 (**g**, left panel) and DU145 (**g**, right panel). Enrichment of RFC promoter region was normalized to the input. IgG was used as a negative control. Mean ± S.D. for three independent experiments is shown. *p < 0.05, **p < 0.01, ***p < 0.001 (Student’s *t*-test).

In line with the potential link of BTF3 with DNA replication and DNA damage repair as shown above, silencing of BTF3 significantly repressed the expression of genes encoding the subunits of the Replication Factor C (RFC) family (Fig. 4a and Supplementary Fig. S4), a five-subunit protein complex involved in the regulation of a variety of important cellular processes including DNA replication and DNA damage response^32,33^. Subsequent quantitative reverse transcription PCR (qRT-PCR) analysis verified that silencing of BTF3 resulted in decreased expression of individual RFC genes in prostate cancer (PC-3 and DU145) and 293 T cells (Fig. 4b). Consistently, DU145 xenograft tumors with inducible knockdown of BTF3 expression revealed significantly reduced expression of the RFC genes when compared to the control group (Fig. 4c). In support of these findings, analyses of TCGA_prostate cancer cohorts yielded a significant association between the expression of BTF3 and RFC subunits (Fig. 4d). Together, these results suggest that BTF3 upregulates the expression of RFC genes.

We next conducted luciferase reporter assays to assess the transcriptional capability of BTF3 on RFC genes in prostate cancer cells. For this, we cloned a DNA sequence between −2500 to +50 relative to each specific RFC gene transcriptional start site into a pGL3-Basic plasmid to yield each respective pGL3-RFC (1, 2, 3, 4 and 5). The reporter gene assay showed that silencing of BTF3 significantly decreased the luciferase activity of pGL3-RFC (1, 2, 3, 4 and 5) in 293 T cells (Fig. 4e). Thus, in line with the qRT-PCR results above, these data indicate that BTF3 may regulate the transcriptional activity of RFC gene promoters. To further investigate whether BTF3, as a transcription factor, could occupy the promoter of the RFC genes, we performed chromatin immunoprecipitation (ChIP) assay to evaluate the binding ability of BTF3 to the RFC gene promoters. We found that the anti-BTF3 antibody, but not the isotype IgG, efficiently retrieved the proximal region of each specific RFC promoter in both 293 T and prostate cancer cells (PC-3 and DU145) (Fig. 4f, g). Collectively, these results support the notion that BTF3 acts as an oncogenic transcription factor to directly upregulate the expression of RFC genes in prostate cancer cells.

**Fig. 5:**
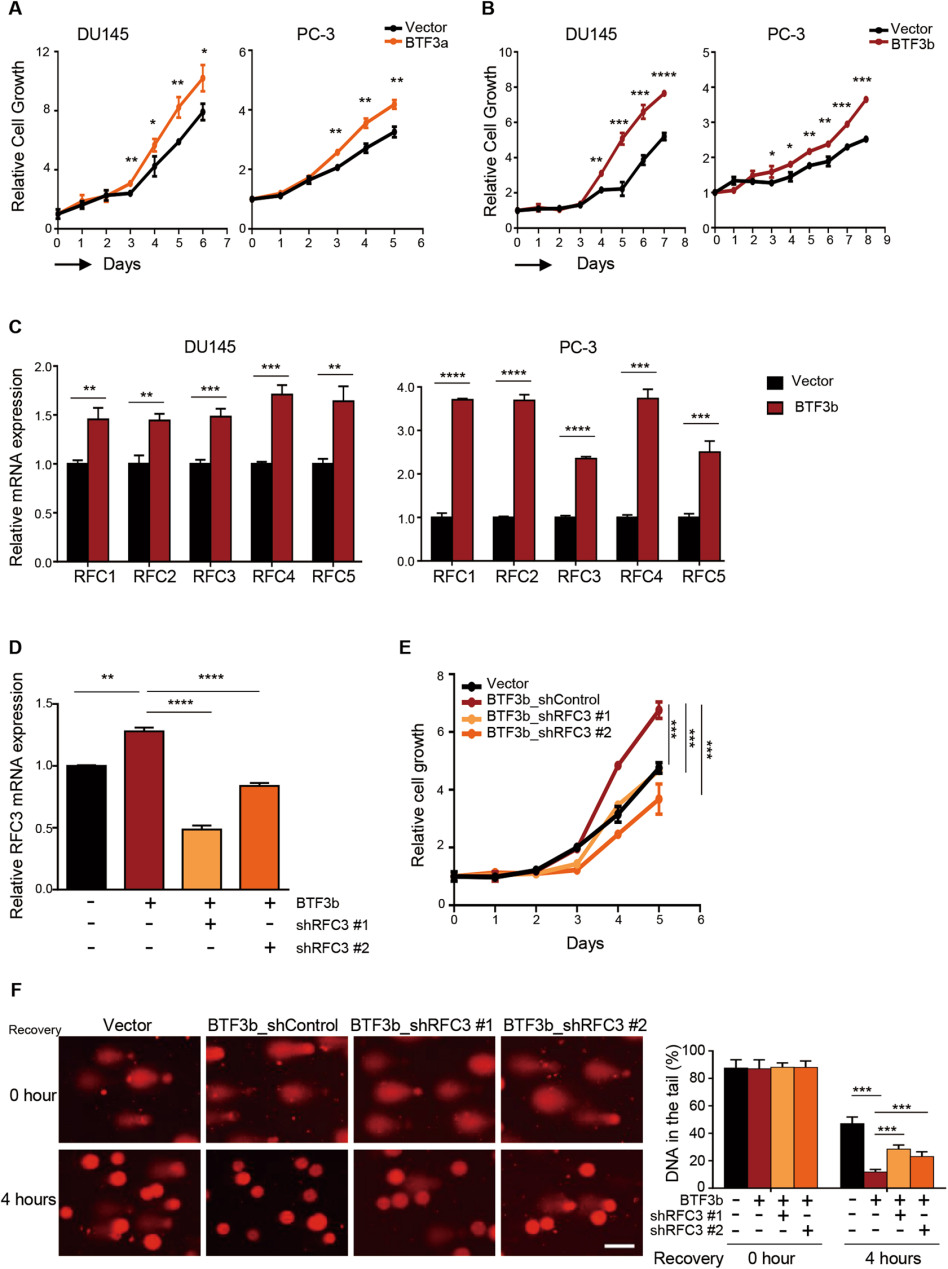
Knockdown of RFC3 partially attenuated the growth advantage and DNA damage repair capability incurred by BTF3b-overexpression. **a**, **b** Relative cell growth of DU145 and PC-3 prostate cancer cells with or without ectopic BTF3a (**a**) or BTF3b (**b**) overexpression. Mean ± S.D. for three independent experiments are shown. *p < 0.05, **p < 0.01, ***p < 0.001, ****p < 0.0001 (Student’s *t*-test). **c** qRT-PCR analysis of the *RFCs* mRNA levels was conducted in DU145 (left panel) and PC-3 (right panel) prostate cancer cells with or without ectopic BTF3b overexpression. **d** qRT-PCR analysis of *RFC3* mRNA levels was conducted in DU145 cells as indicated. *ACTB* was used as an endogenous control. Data are shown as mean ± S.D. from three independent experiments. **p < 0.01, ***p < 0.001, ****p < 0.0001 (Student’s *t*-test). **e** Relative cell growth of prostate cancer cells as in **d**. Data are shown as mean ± S.D. for three independent experiments. ***p < 0.001 (Student’s *t*-test). **f** The effect of RFC3 silencing on the extent of DNA damage in BTF3b-overexpressing DU145 cells was measured by alkaline comet assay. Cells were harvested at the indicated time points after a 30-min treatment with H_2_O_2_ (100 μM). Scale bar, 50 μm. Quantification of DNA in the tail is shown as mean ± SD. ***p < 0.001 (Student’s *t*-test).

We next generated PC-3 and DU145 cells stably overexpressing one of the two BTF3 splicing isoforms, BTF3a or BTF3b^17,18^, and subjected them for further functional characterization. Indeed, ectopic overexpression of either BTF3 isoform resulted in markedly enhanced cell growth (Fig. 5a, b and Supplementary Fig. S5a). However, overexpression of BTF3b, but not BTF3a, induced a significantly increased expression of the RFC genes in DU145 and PC-3 cells (Fig. 5c and Supplementary Fig. S5b). In addition, knockdown of RFC3, one of the RFC subunits, significantly attenuated the growth advantage incurred by BTF3b overexpression (Fig. 5d, e). As RFC subunits play an essential role in DNA damage repair^32,33^, BTF3b overexpressing cells may exhibit higher capacity to repair DNA through inducing the transcription of RFC genes. To test this idea, we conducted comet assays to examine the effect of BTF3b overexpression on the ability of DU145 cells to repair the DNA damage caused by hydrogen peroxide (H_2_O_2_). Compared to the control cells, BTF3b-overexpressing cells exhibited significantly reduced comet tail formation 4 h following the exposure to H_2_O_2_ (Fig. 5f), suggesting that overexpression of BTF3b may renders cells with enhanced DNA damage repair capacity. Moreover, silencing of RFC3 (an RFC subunit) in BTF3b-overexpressing cells resulted in significantly increased amount of DNA in the comet tail (Fig. 5f), supporting a potential role of RFC3 in mediating the effect of BTF3b on DNA damage repair. Together, these results indicate that BTF3b may exert oncogenic activity in prostate cancer through transcriptional regulation of RFC genes and modulation of DNA damage repair capability.

### Overexpression of BTF3b sensitized prostate cancer cells to cisplatin in vitro and in vivo

**Fig. 6:**
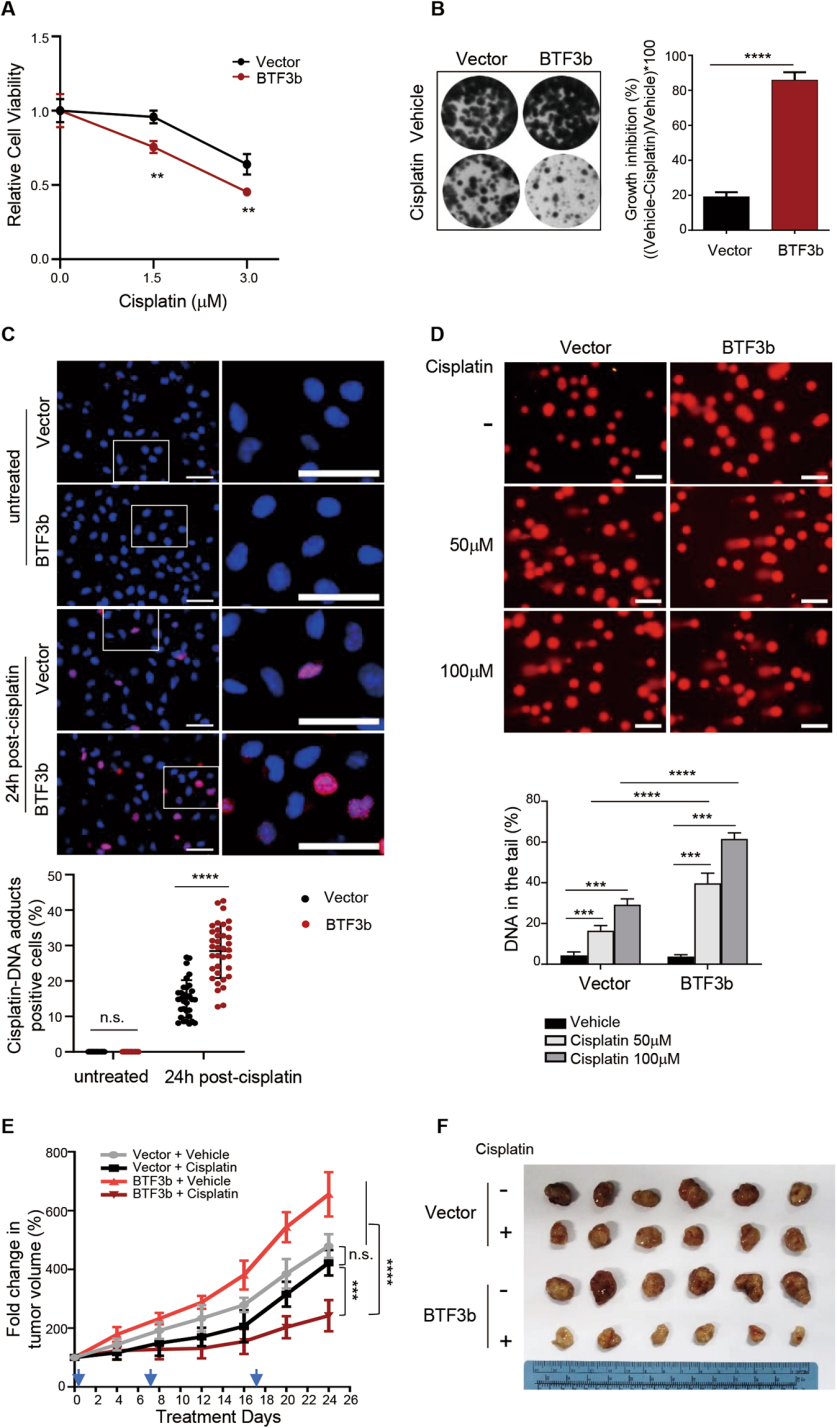
Overexpression of BTF3b enhanced the sensitivity of prostate cancer cells to cisplatin in vitro and in vivo. **a** Cell viability was measured for BTF3b-overexpressing DU145 or control cells treated with or without cisplatin. Cells were exposed to drug treatment for 2 h and then subjected to fresh media for 3 days before MTT assay. Data are shown as mean ± S.D. for three independent experiments. **p < 0.01 (Student’s *t*-test). **b** BTF3b-overexpressing DU145 or control cells were continuously treated with or without cisplatin (0.5 μM) and then subjected to crystal violet assay. Representative images of the plates are shown. Quantification is shown as mean ± S.D. for three independent experiments. ****p < 0.0001 (Student’s *t*-test). **c** Immunofluorescent staining of cisplatin induced DNA-adducts (Red) and DAPI (Blue) in BTF3b-overexpressing DU145 or control cells. Cell were treated with cisplatin (50 μM) for 2 h and cultured for 24 h. Representative images are shown. Quantification is shown as mean ± S.D. from three independent experiments. Scale bar, 100 μm. n.s., not significant, ****p < 0.0001 (Student’s *t*-test). **d** Comet assay was performed to evaluate DNA damage in cells as in **c** treated with or without cisplatin at indicated concentration for 1 h. Quantification of DNA in the tail from three independent experiments is shown as mean ± S.D. Scale bar, 100 μm. Data are shown as mean ± S.D. for three independent experiments. ***p < 0.001, ****p < 0.0001 (Student’s *t*-test). **e** The tumor growth curve of DU145 xenografts expressing BTF3b or control vector treated with cisplatin (5 mg/kg) or vehicle. The arrows indicate the treatment time points. **f** Representative images showed dissected tumors in different treatment groups as indicated at the endpoint. The data are shown as mean ± S.E.M. *n* = 6 for each group. n.s., not significant, ***p < 0.001, ****p < 0.0001 (one-way ANOVA, with Tukey’s multiple comparison tests).

As our data indicated that BTF3 was involved in regulation of DNA replication and DNA damage repair in prostate cancer cells, we next examined the correlation of BTF3 expression with treatment response to cisplatin, a DNA crosslinking agent that causes DNA damage^3–5^. Interestingly, while BTF3 knockdown had little effect on cisplatin sensitivity compared to the control DU145 cells (Supplementary Fig. S6a), overexpression of BTF3b but not BTF3a rendered pronounced drug sensitivity (Fig. 6a, b and Supplementary Fig. S6b–d). However, additional knockdown of RFC3 in BTF3b-overexpressing DU145 cells did not alter cisplatin sensitivity (Supplementary Fig. S6e). As RFC consists of five subunits^32^, our data cannot exclude the possibility that BTF3b expression is associated with cisplatin sensitivity through transcriptional regulation of other RFC components or multiple RFC subunits.

To explore the mechanism by which BTF3b overexpression renders cisplatin sensitivity, we used an anti-cisplatin modified DNA antibody to quantitatively detect the accumulation of cisplatin-DNA adducts in cells with or without BTF3b overexpression in response to cisplatin treatment. Indeed, 24 h post cisplatin treatment, BTF3b- overexpressing DU145 cells exhibited significantly greater accumulation of modified DNA than the control cells (Fig. 6c). Accordingly, cisplatin treatment induced significantly increased DNA damage in BTF3b overexpressing cells as assessed by the amounts of DNA in the comet tails of DU145 cells (Fig. 6d).

We next extended these findings to investigate the effect of BTF3b overexpression on cisplatin sensitivity in vivo. For this, we established xenograft models of BTF3b-overexpressing DU145 cells in male nude mice and subjected the tumor-bearing mice to cisplatin treatment. We found that BTF3b-overexpressing tumors grew significantly faster than the control tumors (Fig. 6e, f), consistent with the notion that BTF3 confers gain-of-function activity in prostate cancer. More importantly, while cisplatin treatment led to substantially attenuated growth of BTF3b-overexpressing tumors, it had only limited inhibitory effect on the control DU145 tumors (Fig. 6e, f). Together, these results suggest that BTF3b overexpression renders prostate tumors more responsive to cisplatin treatment.

## Discussion

Various transcription factors have been reported to play key roles in tumor growth, disease progression, drug resistance and metastases in prostate cancer^11,13,15^. Here we have uncovered a novel role of BTF3 in prostate tumorigenesis through transcriptional upregulation of RFC subunits involved in DNA replication and repair processes. Platinum compounds have been tested in multiple clinical trials and generate only moderate response in molecularly unselected patients with advanced prostate cancer. Interestingly, our data suggest that BTF3 overexpression sensitizes prostate cancer cells to the DNA damaging agent cisplatin and may serve as a potential predictive marker for the selection of patient for platinum-based treatment.

In the current study, we first showed that knockdown of BTF3 significantly attenuated the growth, migration and invasion of prostate cancer cells, validating the oncogenic effects of BTF3 in prostate cancer. Subsequent integrative analyses of transcriptomics and clinical data directed our attention to the potential link of BTF3 expression with DNA replication and a number of DNA repair pathways including nucleotide excision repair, mismatch repair and base excision repair. Among all those genes downregulated due to BTF3 silencing, we chose to focus on RFC subunit-encoding genes as the RFC complex is known to be involved in DNA replication and repair processes^32,33^. Indeed, our work demonstrates that BTF3 transcriptionally regulates the expression of genes encoding all five RFC subunits, and that knockdown of RFC3, one of the RFC subunits, at least in part attenuates the growth advantage and DNA damage repair capability conferred by BTF3 overexpression. Collectively, BTF3-mediated transcriptional regulation of RFC subunits may account for the oncogenic action of BTF3 in prostate cancer.

Our work showed that inhibition of BTF3 resulted in substantial DNA damage as evidenced by increased comet tails, γH2AX nuclear foci and G2/M arrest. Homologous recombination repair deficiency is known to confer sensitivity to cisplatin, a DNA damaging chemotherapeutic agent^34–36^. However, whether nucleotide excision repair pathway inactivation is directly correlated to cisplatin sensitivity has remained largely elusive^35,37–39^. In our study, while BTF3 is involved in the regulation of DNA replication and repair processes, silencing of BTF3 did not render prostate cancer cells more responsive to cisplatin (Supplementary Fig. S6a). Notably, a recent study by Pillay et al has demonstrated the synthetic lethal relationship of inhibition of DNA replication factors, especially those involved in replication fork stability including RFC2, with inhibition of PARG (an enzyme that degrades PAR chains at DNA damage sites)^40^. If this scenario also holds true in prostate cancer, our study may warrant further investigation of whether BTF3 silencing-induced DNA replication vulnerability sensitizes cells to PARG inhibition, a potential new treatment strategy that can be extended to treat prostate cancer patients.

BTF3 has two splicing isoforms, BTF3a and BTF3b, both of which confer transcriptional activity in ER-positive breast cancer cells^19,20^. Our study reveals that BTF3b but not BTF3a transcriptionally regulates the expression of genes encoding RFC subunits. While ectopic overexpression of either BTF3a or BTF3b significantly promotes the growth of prostate cancer cells, overexpression of BTF3b but not BTF3a sensitizes prostate cancer cells to cisplatin treatment. It is worth noting that the BTF3-specific shRNA sequence used in this study does not reside within the N-terminal region that differentiates the BTF3a gene sequence from that of BTF3b^18^ (Materials and methods). It is therefore unlikely to dissect the differential biological roles of BTF3a and BTF3b simply by the gene silencing approach. However, we still cannot rule out the possibility that BTF3a also exhibits transcriptional activity to regulate alternative genes promoting prostate tumorigenesis.

Up to 20–30% of advanced prostate cancer has been identified with DNA repair defects^41,42^, which would theoretically benefit from platinum-based therapy. However, platinum compounds have exhibited limited anti-tumor activity in molecularly unselected prostate cancer patients, and there are only a few cases of complete treatment responses reported in the clinical trials^7–10^. Hence there remains an unmet medical need to identify predictive biomarkers that allow molecular selection of prostate cancer patients most likely to gain clinical benefits from platinum-based therapy. Interestingly, our data showed that overexpression of BTF3b sensitized cells to cisplatin in in vitro assays as evidenced by significantly increased cisplatin-DNA adducts and enhanced comet tail formation. Concordantly, BTF3b overexpression resulted in increased, rather than decreased, cisplatin sensitivity in a DU145 prostate tumor xenograft model. Indeed, our findings regarding the association of BTF3 expression and cisplatin sensitivity phenocopied the effect of ERCCs, nucleotide excision repair proteins, on cisplatin responsiveness^37,43^. Future studies would be necessary to understand the underlying mechanism why overexpression of certain genes that regulate DNA repair processes, *e.g*. ERCCs and BTF3b, confers sensitivity to cisplatin. BTF3 has been recently found to sustain cancer stem-like traits of prostate cancer via stabilization of BMI1^29^. Moreover, this recent work reveals that BTF3 expression may predict poor prognosis and act as a risk stratification marker of prostate cancer patients. Our findings that BTF3 promotes tumorigenesis through transcriptional upregulation of Replication Factor C (RFC) subunits thus add a new perspective on the potential oncogenic roles of BTF3 in prostate cancer. More importantly, our work highlights the potential of BTF3 overexpression as a predictive marker to stratify the right patients for cisplatin or novel mechanism-based therapeutics.

## Materials and methods

### Cell culture and reagents

The human prostate cancer cell lines PC-3 and DU145, and human embryonic kidney cell line HEK293T were purchased from American Type Culture Collection (ATCC, Virginia, USA). Cells were maintained in culture medium (PC-3 in RPMI-1640, DU145 and HEK293T in DMEM) supplemented with 10% fetal bovine serum (FBS, Biological Industries, Kibbutz Beit-Haemek, Israel) and 100 units/ml penicillin/streptomycin (Gibco, Massachusetts, USA) at 5% CO_2_ and 37 °C in a humidified incubator. All cell lines were authenticated by short tandem repeat profiling (STR) and tested periodically for mycoplasma contamination. Cisplatin was purchased from MedChemExpress (MCE, New Jersey, USA), puromycin and doxycycline were purchased from Sigma (Missouri, USA), and Blasticidin was purchased from Invitrogen (Massachusetts, USA). siRNAs were custom synthesized from GenePharma (Shanghai, China). Cells were transfected with on-target (siBTF3 #1, 5′-GCCGAAGAAGCCUGGGAAUCA-3′) or non-targeting control siRNA (siNC, 5′-UUCUCCGAACGUGUCACGUTT-3′) using Lipofectamine 2000 (Invitrogen, Massachusetts, USA) according to the manufacturer’s protocols.

To achieve BTF3 knockdown, specific oligonucleotides were cloned into lentiviral shRNA expression vectors (pLKO.1 puro (Addgene # 8453) for straight knockdown and Tet-on-pLKO puro (Addgene # 21915) for doxycycline-inducible knockdown). The following primer sequences were used:

shBTF3 #1: Fw 5′-CCGGGCCGAAGAAGCCTGGGAATCACTCGAGTGATTCCCAGGCTTCTTCGGCTTTTTG-3′, Rv 5′-AATTCAAAAAGCCGAAGAAGCCTGGGAATCACTCGAGTGATTCCCAGGCTTCTTCGGC-3′.

shBTF3 #2: Fw 5′-CCGGCCCAGCATCTTAAACCAGCTTCTCGAGAAGCTGGTTTAAGATGCTGGGTTTTTG-3′, Rv 5′-AATTCAAAAACCCAGCATCTTAAACCAGCTTCTCGAGAAGCTGGTTTAAGATGCTGGG-3′.

For BTF3a overexpression, human full-length BTF3a cDNA was cloned into pLenti6-V5-Topo mCherry (Addgene #128062) by using the following primer sequences:

BTF3a: Fw 5′-CGGATCCGCCATGCGACGGACAGGCG-3′, Rv 5′-CCGCTCGAGTCAGTTTGCCTCATTCTTGGAAGCCTC-3′.

pLX304-Blast-V5-BTF3b was purchased from Dharmacon (Colorado, USA).

### Western blot analysis

Cells were lysed in RIPA buffer supplemented with protease and phosphatase inhibitors (Thermo Scientific, Massachusetts, USA). The primary antibody rabbit anti-human BTF3 Ab (ab203517) was purchased from Abcam (Massachusetts, USA), mouse anti-V5-tag Ab (66007-1-Ig) was purchased from Proteintech (Illinois, USA), and mouse anti-Vinculin Ab (V9131) was from Sigma-Aldrich (Missouri, USA). Fluorescent-labeled secondary antibodies to mouse IgG and rabbit IgG were purchased from Li-COR Biosciences (Nebraska, USA). Western blots were imaged using Odyssey Infrared Imaging System (Li-COR Biosciences, Nebraska, USA).

### Quantitative real-time PCR

Total RNA was isolated using NucleoZOL (Macherey-Nagel, Dueren, Germany) and cDNA was synthesized by reverse transcription reaction using the PrimeScript^TM^ RT Master Mix (Perfect Real Time) (TAKARA, Shiga, Japan) according to the manufacturer’s instructions. Quantitative real-time PCR was performed using SYBR Green PCR Master Mix (Applied Biosystems, Massachusetts, USA) on a StepOnePlus^TM^ RealTime PCR System (Life Technologies, Massachusetts, USA). The relative gene expression levels were calculated by the 2^-ΔΔCt^ method and normalized to *ACTB*. The following primers were used:

*BTF3*: Fw 5′-GCCAGTCTCCTTAAACTAGTCAG-3′, Rv 5′-TTTCACCATTACAGGCCATGCT-3′. *RFC1*: Fw 5′-CCATCGCCAAGCAATTACAG-3′, Rv 5′-GGTTCTTCATCCAACATGGC-3′. *RFC2*: Fw 5′-GGCAACATCTTTCGAGTGTG-3′, Rv 5′-GAGTTCACTCCTTCCGCTAT-3′. *RFC3*: Fw 5′-GCCCTGCTTATGTGTGAAGC-3′, Rv 5′-GCATTTGCAGTCTCCCTCAG-3′. *RFC4*: Fw 5′-CCACCCGATTCTGTCTTATC-3′, Rv 5′-CTAGTAATCGCTGCTGTTGA-3′. *RFC5*: Fw 5′-GTCAGACATTGCCAACATCC-3′, Rv 5′- AGGATATCATGCAGTGCCAA-3′. *ACTB*: Fw 5′-CATGTACGTTGCTATCCAGGC-3′, Rv 5′-CTCCTTAATGTCACGCACGAT-3′.

### Clonogenic survival assay

Cells were fixed and stained with 0.5% crystal violet followed by washing with PBS before drying. Then the bound crystal violet was dissolved by 10% acetic acid solution. The optical density (OD) of bound crystal violet was measured at 590 nm using xMark Microplate Spectrophotometer (Bio-Rad, California, USA).

### Cell viability assay

Cell viability after drug treatment was measured with MTT assay (Sigma-Aldrich, Missouri, USA) according to the manufacturer’s guidelines. The optical density (OD) was measured at 490 nm on xMark Microplate Spectrophotometer (Bio-Rad, California, USA).

### Three-dimensional (3D) spheroid assay

Three-dimensional (3D) spheroid assay was performed as described previously^20^. In brief, cells were seeded on plates precoated with 50% Matrigel (BD Biosciences, California, USA) plus 50% serum-free medium. Cells were maintained in culture media supplemented with 2% FBS and 2% Matrigel with or without indicated drug that was replaced every 3 days. Three-dimensional cell cultures were imaged by inverted phase-contrast microscope (Leica Microsystems, Wetzlar, Germany) and the 3D spheroid areas were quantified with ImageJ software.

### Migration assay

Cell migration was evaluated by wound-healing assay as previously described^44^. Briefly, cells were seeded into 12-well plates and scratched with sterile tips when cells were grown to form a confluent monolayer. The cells were washed three times with PBS to remove cell debris and fresh culture media was added. The wounds were photographed with inverted phase-contrast microscope (Olympus corporation, Tokyo, Japan) and the width of each scratch wound was measured using the ImageJ software.

### Invasion assay

Cell invasion was evaluated by transwell assay (Corning, USA) according to the manufacturer’s recommendations. Briefly, the invasion chambers were loaded with Matrigel (BD Biosciences, California, USA). Single cell suspension in serum-free medium was seeded into the chamber, and culture medium containing 10% FBS was added into the well plates. After incubation for 24 h, the non-invading cells were gently removed, and the invading cells were fixed, stained with crystal violet solution and photographed with inverted phase-contrast microscopy (Olympus corporation, Tokyo, Japan), and the number of invading cells was counted using the ImageJ software.

### Flow cytometry analysis

For cell cycle analysis, cells were harvested using 0.25% trypsin (EDTA free) and gently fixed overnight using ice-clod 75% ethanol. Cells were then stained with Propidium Iodide (PI)/RNase solution (50 mg/ml Propidium Iodide, 0.1 mg/ml RNase and 0.2% Triton X-100) at dark for 30 min. For apoptosis assay, cells were harvested according to the manufacturer’s instruction of Annexin V/PI Apoptosis Detection Kit (Dojindo Molecular Technologies, Inc., AD10, Kumamoto, Japan). Briefly, cells were harvested with 0.25% trypsin (EDTA free), washed with 1X staining buffer, and stained with Annexin V solution/PI solution in dark for 15 min. The stained cells were analyzed on BD FACSCanto™ II (BD Biosciences, California, USA).

### RNA sequencing and data analysis

Total RNA was isolated from cells using Trizol Reagent (Thermo Fisher, Massachusetts, USA) according to the manufacturer’s instruction. RNA sequencing was carried out on Illumina HiSeq platform by Novogene Corporation (Beijing, China). The sequencing libraries were created using NEBNext® Ultra^TM^ RNA Library Prep Kit for Illumina® (NEB, Massachusetts, USA) according to the manufacturer’s instructions. FPKM (expected number of Fragments Per Kilobase of transcript sequence per Millions base pairs sequenced) was used to estimate the gene expression levels. Heat map was drawn using genes with adjusted p-values less than 0.05 with ‘pheatmap’ package. ClusterProfiler R package was used to test the statistical enrichment of differential expression genes in KEGG pathways. Gene Set Enrichment Analysis (GSEA) was performed across the Molecular Signatures Database (MSigDB) using the JAVA program to identify the molecular pathways correlated to BTF3 expression in PC-3 cells. The RNA-seq dataset was deposited to the Gene Expression Omnibus (GEO) with accession number GSE139528.

### Immunofluorescence assay

Immunofluorescence staining was performed as previously described^45^. Briefly, cells were attached to glass slides and maintained in culture medium. Cells were subjected to fixation with 4% formaldehyde (for the detection of γH2AX) or 70% ethanol (for the detection of cisplatin-DNA adducts), permeabilization with 0.2% Triton X-100, followed by blocking with 5% BSA. Subsequently, cells were incubated with anti-γH2AX (Cell Signaling Technology, CST, #2577, Massachusetts, USA) or anti-cisplatin modified DNA antibody (Abcam, ab103261, Massachusetts, USA) at 4 °C overnight. Lastly, cells were stained with fluorescence-conjugated secondary antibodies and DAPI solution (Sigma-Aldrich, Missouri, USA), and then photographed with Leica fluorescence microscope (Leica Microsystems, Wetzlar, Germany).

### Chromatin immunoprecipitation analysis

Chromatin immunoprecipitation (ChIP) assay was performed as previously described^46^. Briefly, after cross-linked with 1% formaldehyde solution, quenched with 125 mM glycine and sonicated (Qsonica, Q125, Connecticut, USA) on ice, cell lysates were immunoprecipitated at 4 °C for 4 h with anti-BTF3 antibody (Abcam, ab203517, Massachusetts, USA) or isotype rabbit IgG (Santa Cruz, sc-2027, California USA), followed by incubation with protein G magnetic beads (Invitrogen, 11203D, Massachusetts, USA) for 2 h. The ChIP-enriched DNA was eluted and subjected to quantitative real time PCR using promoter-specific primers as follows:

ChIP-RFC1: Fw 5′-GTGAGGCCCTGTTAATAAGT-3′, Rv: 5′-AACGCCTATTGTTGTACGTG-3′.

ChIP-RFC2: Fw 5′-AGCCGAGAATTCCCTGATAAT-3′, Rv 5′-CAGATTGCTCTGAATGTCCTAGT-3′.

ChIP-RFC3: Fw 5′-GGTGGTGTGGATAATACCTTACC-3′, Rv 5′-CTTTCACCAAGGTGCCTCTTAC-3′.

ChIP-RFC4: Fw 5′-TGCATGGTCTTCTCTCTCCA-3′, Rv 5′-TGTGCTTTAAGGGCAGAGAC-3′.

ChIP-RFC5: Fw 5′-CCATCACATACTTGCCACTG-3′, Rv 5′-GGGTCAGCAGTGTTCTGAT-3′.

### Luciferase reporter assay

The proximal promoter sequences of RFC family genes were amplified and cloned into the pGL3-Basic plasmid, respectively. siBTF3 or siNC transfected 293 T cells were seeded into 96-well plates, and transfected with firefly-luciferase plasmids (pGL3-RFC or pGL3-Basic control plasmid) and pRL-TK plasmids (used as normalization controls) using Lipofectamine 2000 (Invitrogen, Massachusetts, USA) according to the manufacturer’s protocol. After transfection for 48 h, luciferase activities were quantified using Dual-Luciferase Reporter Assay Kit (Promega, E2920, Wisconsin, USA) according to the manufacturer’s instruction on Enspire 2300 multilabel reader (Perkin Elmer, Connecticut, USA). The primers used in the assay are listed as follows:

pGL3- RFC1: Fw 5′-CGGCTAGCAGAAGTTACAGTCTCCTGACT-3′, Rv 5′-CGAGATCTATCGAGGCTCAGGATCCATTC-3′.

pGL3-RFC2: Fw 5′-CGGCTAGCCATGGTTCACTGTGGCTTCAAC-3′, Rv 5′-CGAGATCTGCCACTCTCGATCCATGTCCG-3′.

pGL3-RFC3: Fw 5′-CGGCTAGCTACAATTACTATATGATTGCC-3′, Rv 5′-CGAGATCTCTCTTCAAATAGTGCCTCTC-3′.

pGL3-RFC4: Fw 5′-CGGCTAGCGGTGACCAGATAGATAACCTG-3′, Rv 5′-CGAGATCTTCCAGCAGGTTACCAGTTAGC-3′.

pGL3-RFC5: Fw 5′-CGGCTAGCGGCACTGTCACATCACAAGC-3′, Rv 5′-CGAGATCTAACAGAACGGACGAGTCTGG-3′.

### Comet assay

DNA damage was evaluated by alkaline comet assay as previously described^45^. Briefly, cells were lysed in alkaline lysis buffer, electrophoresed in running buffer, neutralized with PBS, and stained with Gold View (Coolaber, # SL2140-1ML, Beijing, China). Finally, cells were photographed with Leica fluorescence microscope (Leica Microsystems, Wetzlar, Germany) and the percentage of DNA in tails was served as a quantitative measure of DNA damage using CASP (Comet Assay Software Project Lab) software.

### Bromodeoxyuridine incorporation assay

Cells were synchronized by serum starvation and incubated in culture medium supplemented with BrdU (Sigma-Aldrich, Missouri, USA) for 2 h, and fixed in 75% ice-cold ethanol overnight. Then cells were denatured in DNA denaturation buffer (1.5 N HCl and 0.5% Triton X-100), stained with FITC-conjugated BrdU antibody (Invitrogen, MD5401, Massachusetts, USA) and Propidium Iodide/RNase solution (10 μg/ml Propidium Iodide, 0.1 mg/ml RNase and 0.1% NP-40). Samples were analyzed on BD FACS Canto™ II (BD Biosciences, California, USA).

### Immunohistochemistry

The immunohistochemistry assay was performed on the human prostate cancer tissue arrays purchased from Outdo Biotech Co., Ltd. (Shanghai, China). 55 adjacent noncancerous tissues and 82 human primary prostate cancer tissues were stained with primary antibody against BTF3 (Abcam, ab203517, Massachusetts, USA). A Histo-score (H score) was calculated based on the staining intensity and percentage of stained cells. The intensity score was defined as follows: 0, no appreciable staining in cells; 1, weak staining in cells comparable with stromal cells; 2, intermediate staining; 3, strong staining. The fraction of indicated degree staining cells was scored as 0–100%. H score is a mean of staining intensity score and weighted by the fraction.

### In vivo mouse xenograft study

Animal studies were performed under the approval of the Animal Care and Use Committee of Dalian Medical University. 6-week-old Balb/c nude male mice were purchased from Beijing Vital River Laboratory Animal Technology Co., Ltd, (Beijing, China) and maintained in a pathogen-free environment. 5 × 10^6^ cells were suspended in PBS/Matrigel (BD Biosciences, California, USA) and subcutaneously injected into the flanks. When tumors reached average volume of 200 mm^3^, mice were treated with doxycycline (2 mg/ml in drinking water) or cisplatin (5 mg/kg in PBS, intraperitoneal injection). Tumor volumes were measured every two days with calipers and calculated using the following formula: tumor volume = (length × width^2^)/2.

### Statistical analysis

The data obtained from in vitro and in vivo studies were statistically analyzed by the Student’s *t*-test, Mann–Whitney test, or the one-way ANOVA with Tukey’s multiple comparison tests as indicated using GraphPad Prism software. *p*-value < 0.05 was considered to be statistically significant.

## Supplementary information

Supplementary Figure 1

Supplementary Figure 2

Supplementary Figure 3

Supplementary Figure 4

Supplementary Figure 5

Supplementary Figure 6

Supplementary Figure legends
