# Supplementary figures and images for: BTF3 confers oncogenic activity in prostate cancer through transcriptional upregulation of Replication Factor C
Source: Cell Death Dis. 2021 Jan 5;12(1):12. doi: 10.1038/s41419-020-03348-2 (PMC7791038; doi:10.1038/s41419-020-03348-2)

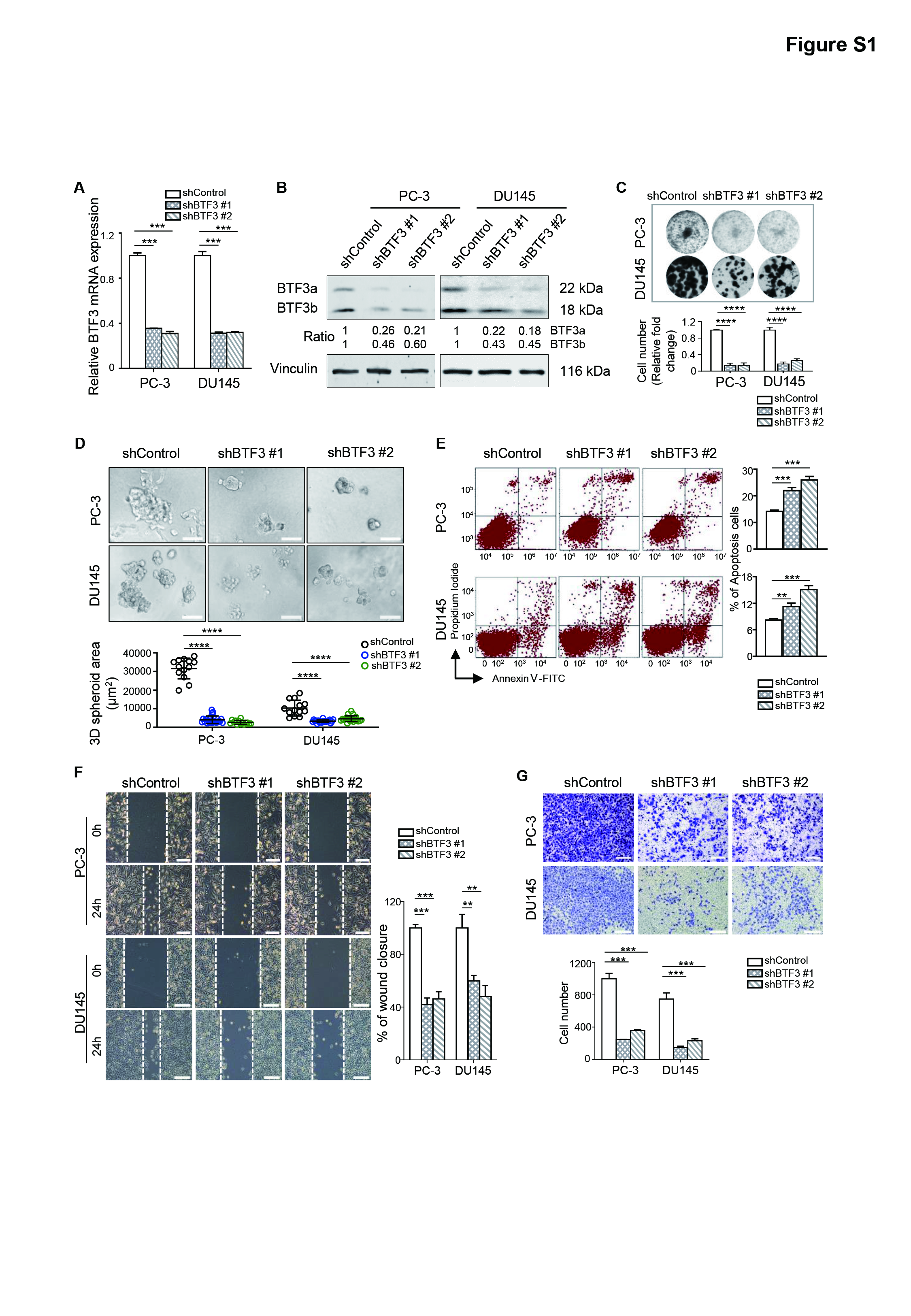

Supplement: Supplementary file 1 — Supplementary Figure 1 [file 41419_2020_3348_MOESM1_ESM.tif]

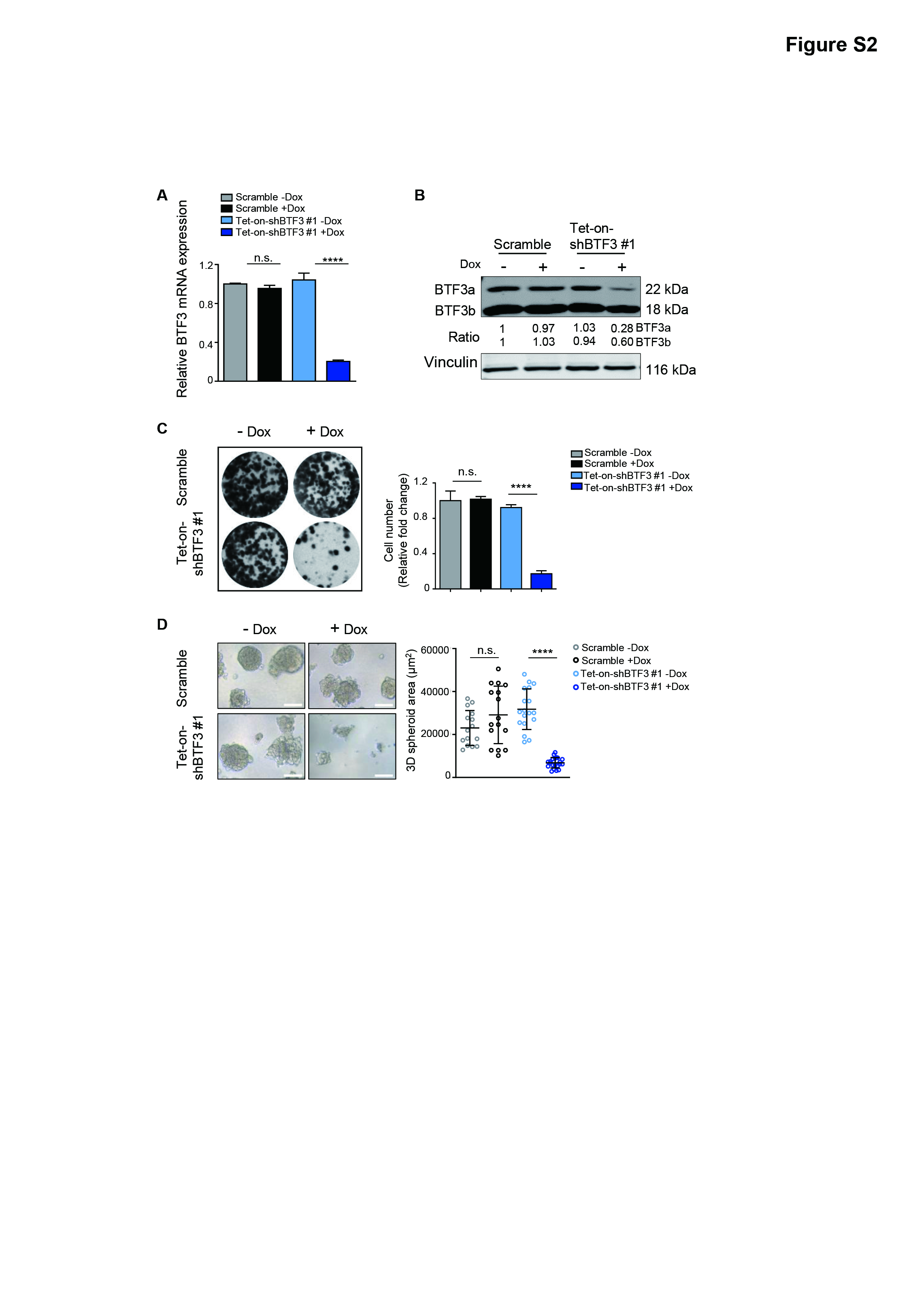

Supplement: Supplementary file 2 — Supplementary Figure 2 [file 41419_2020_3348_MOESM2_ESM.tif]

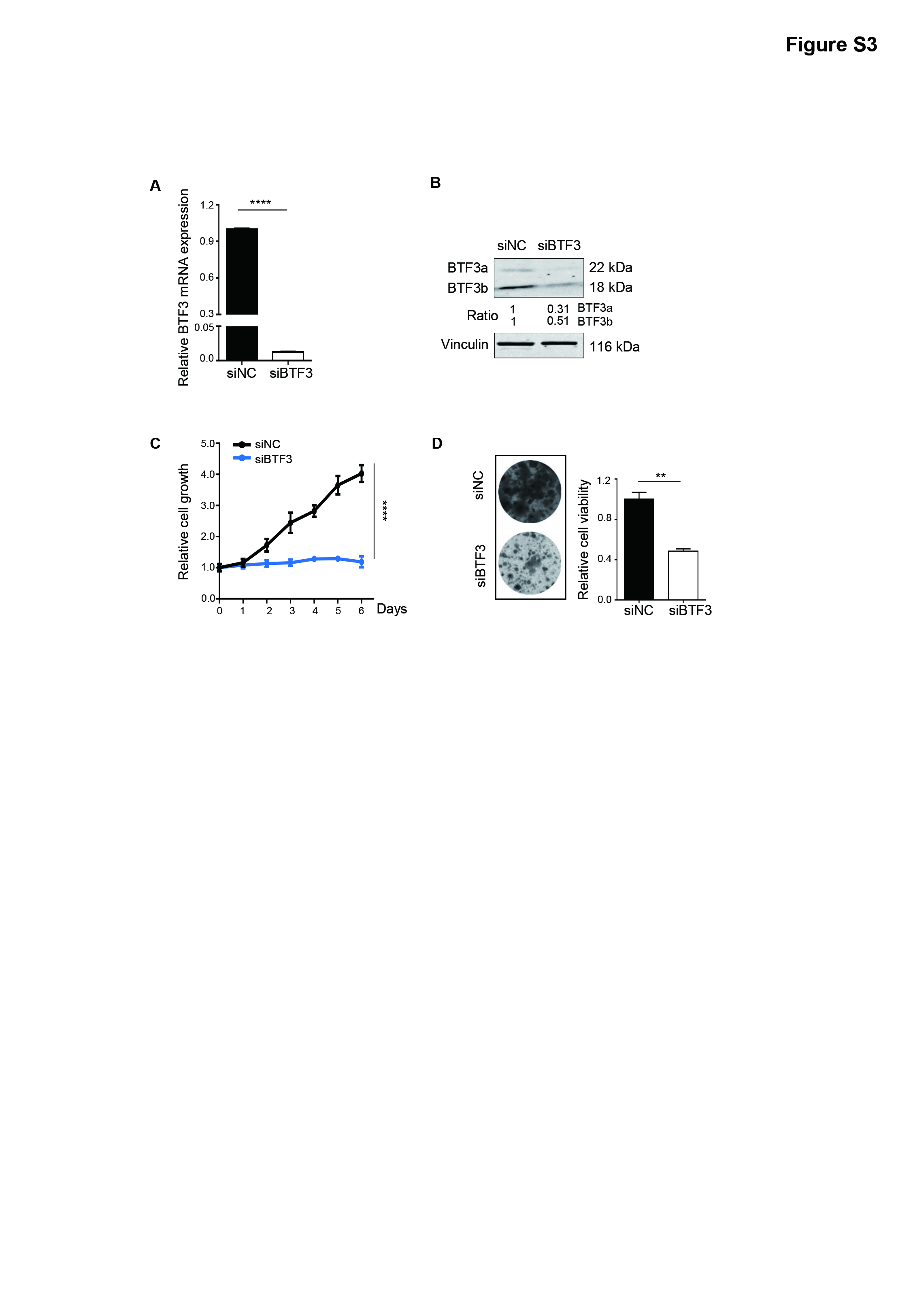

Supplement: Supplementary file 3 — Supplementary Figure 3 [file 41419_2020_3348_MOESM3_ESM.tif]

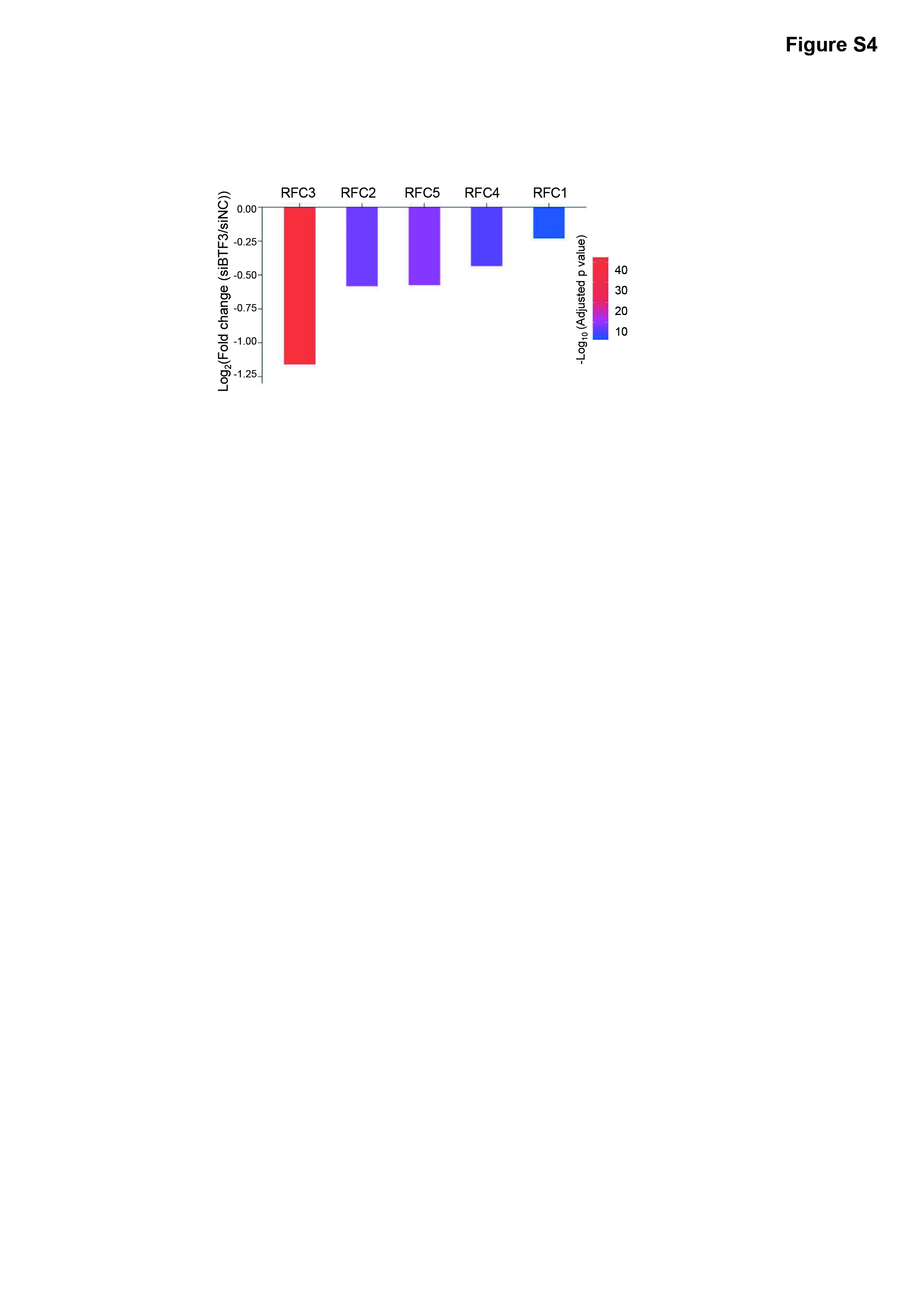

Supplement: Supplementary file 4 — Supplementary Figure 4 [file 41419_2020_3348_MOESM4_ESM.tif]

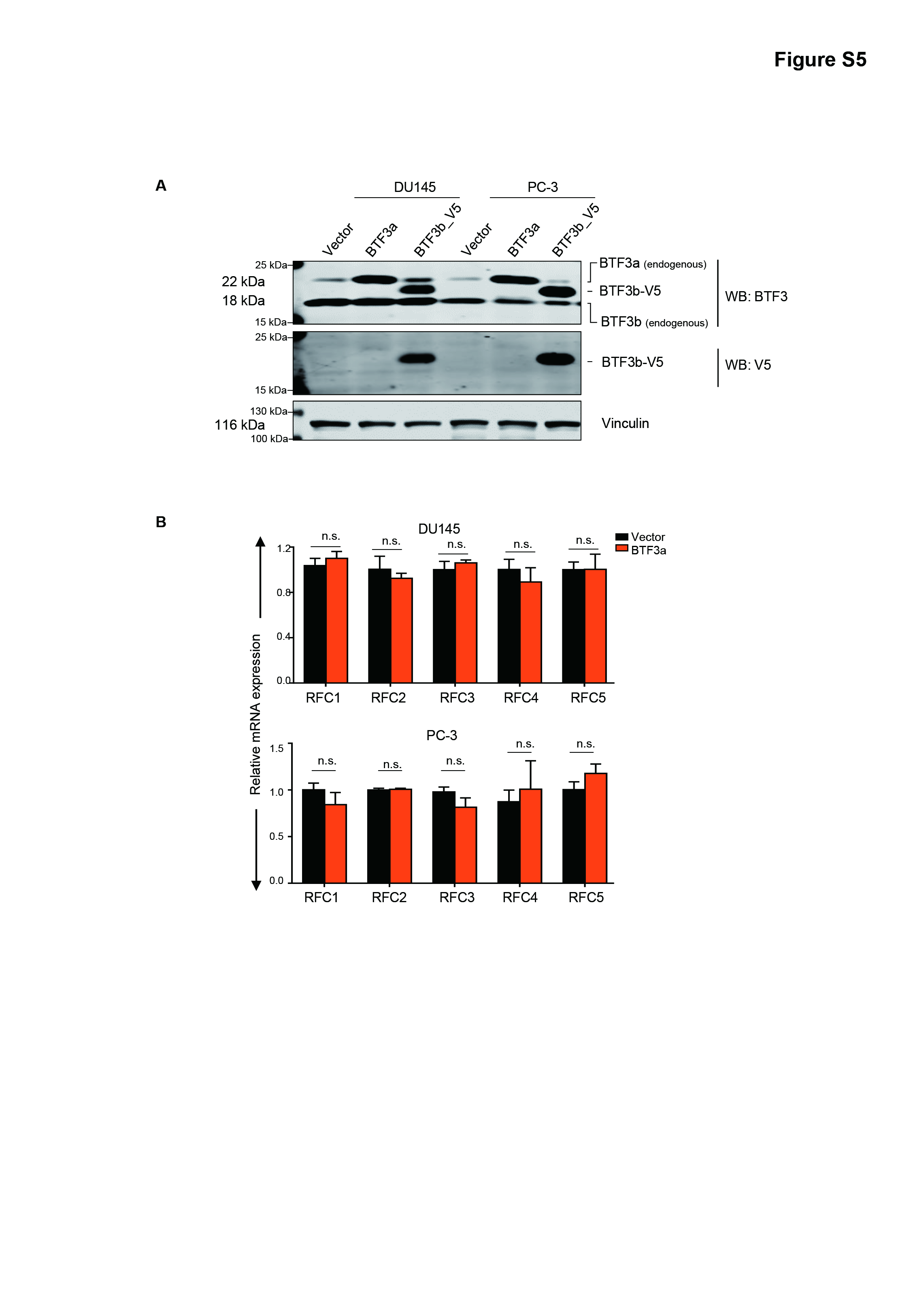

Supplement: Supplementary file 5 — Supplementary Figure 5 [file 41419_2020_3348_MOESM5_ESM.tif]

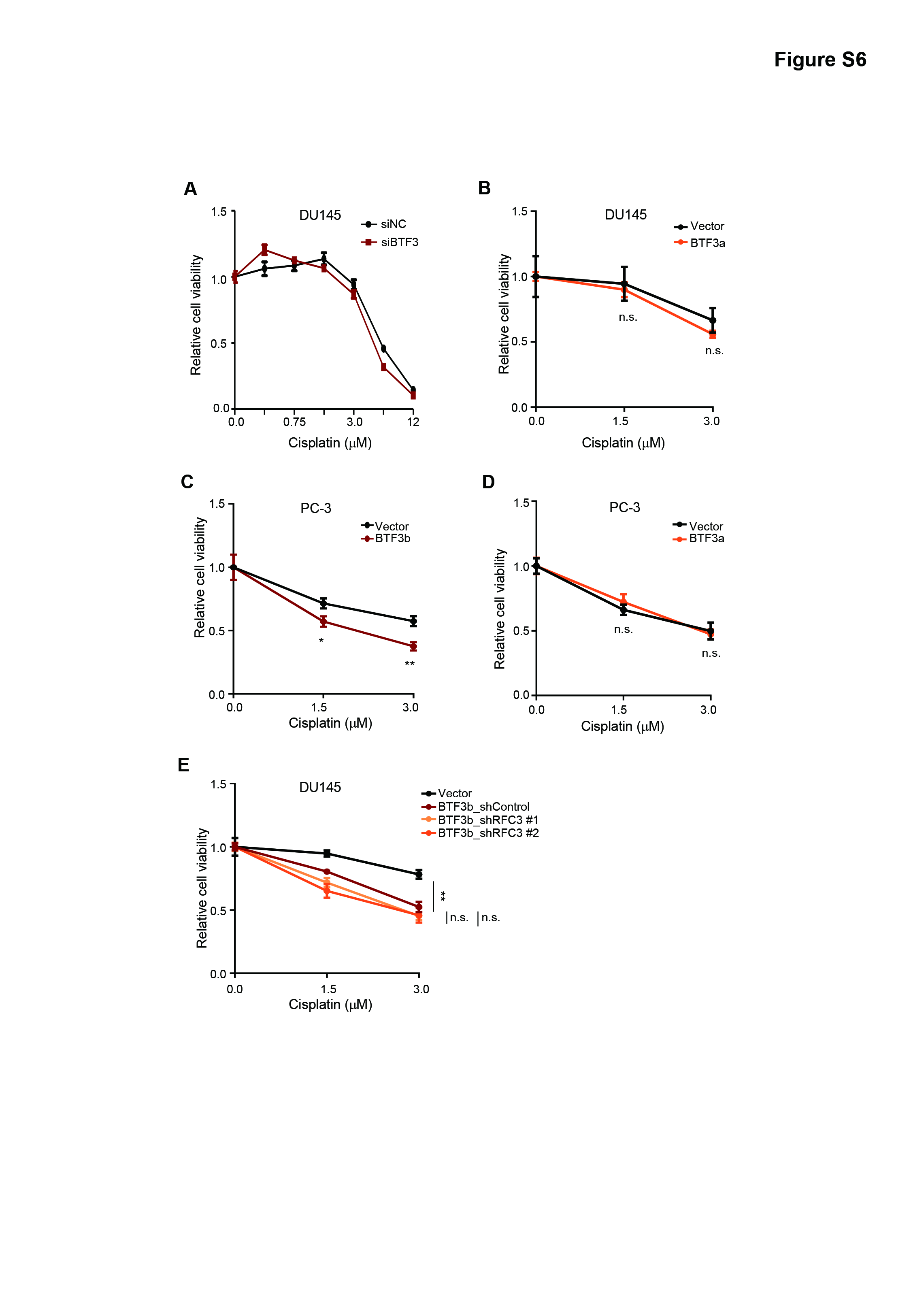

Supplement: Supplementary file 6 — Supplementary Figure 6 [file 41419_2020_3348_MOESM6_ESM.tif]
