# Supplementary material for: BTF3 confers oncogenic activity in prostate cancer through transcriptional upregulation of Replication Factor C
Source: Cell Death Dis. 2021 Jan 5;12(1):12. doi: 10.1038/s41419-020-03348-2 (PMC7791038; doi:10.1038/s41419-020-03348-2)
Supplement: Supplementary file 7 — Supplementary Figure legends [file 41419_2020_3348_MOESM7_ESM.pdf]

Supplementary Figure 1. The effects of shRNA-induced BTF3 knockdown on the growth of prostate cancer cells. a qRT-PCR analysis of the *BTF3* mRNA levels was conducted in PC-3 and DU145 cells with or without BTF3-knockdown. *ACTB* was used as an endogenous control. **b** Western blot analysis of BTF3a and BTF3b protein abundance in BTF3-knockdown PC-3 and DU145 cells. Vinculin was used as a loading control. The quantification of protein abundance is shown. **c** The clonogenic formation assay for cells as in (**a**). Representative images and quantification of the plates are shown. **d** Prostate cancer cells as in (**a**) were cultured in 3D Matrigel. Representative images and the quantitation of 3D spheroids areas are shown. Scale bar, 100 μm. **e** Flow cytometric analysis of Annexin V/PI (propidium iodide) stained BTF3-knockdown PC-3 and DU145 cells was conducted to evaluate apoptosis. **f** The cell migration potential of PC-3 and DU145 with or without BTF3-knockdown was determined using wound-healing assay. Representative images and quantification of the wound areas are shown. Scale bar, 200 μm. **g** The cell invasion potential of PC-3 and DU145 cells with or without BTF3-knockdown was analyzed using Transwell invasion assay. Representative images and quantification of invading cells are shown. Scale bar, 200 μm. Data are shown as mean ± S.D. for three independent experiments (** $p < 0.01$, *** $p < 0.001$, **** $p < 0.0001$ by student's *t*-test).

Supplementary Figure 2. The effects of doxycycline-induced shRNA-mediated BTF3 knockdown on the growth of prostate cancer cells. a qRT-PCR analysis of the *BTF3* mRNA levels was conducted in DU145-Tet-On-shBTF3 or control cells treated with or without doxycycline. *ACTB* was used as an endogenous control. **b**

Western blot analysis of BTF3a and BTF3b protein abundance in DU145-Tet-On-shBTF3 or control cells treated with or without doxycycline. Vinculin was used as a loading control. The quantification of protein abundance is shown. **c** The clonogenic formation assay for cells as in (**a**) treated with or without doxycycline. Representative images and quantification of the plates are shown. **d** DU145-Tet-On-shBTF3 cells were cultured in 3D Matrigel and treated with or without doxycycline. Representative images and the quantitation of 3D spheroids areas are shown. Scale bar, 100 μm. Dox, doxycycline, 0.5 μg/ml. Data are shown as mean ± S.D. for three independent experiments (n.s., not significant, **** $p < 0.0001$ by student's *t*-test).

Supplementary Figure 3. The effects of siRNA-induced BTF3 knockdown on the growth of prostate cancer cells. a qRT-PCR analysis of the *BTF3* mRNA levels was conducted in PC-3 cells transfected with siNC (Non-targeting Control) or siBTF3. *ACTB* was used as an endogenous control. **b** Western blot analysis of BTF3a and BTF3b protein abundance in PC-3 cells transfected with siNC or siBTF3. Vinculin was used as a loading control. The quantification of protein abundance is shown. **c** Relative cell growth of cells as in (**a**). **d** The clonogenic formation assay for cells as in (**a**). Representative images and quantification of the plates are shown. Data are shown as mean ± S.D. for three independent experiments (** $p < 0.01$, **** $p < 0.0001$ by student's *t*-test).

Supplementary Figure 4. Knockdown of BTF3 resulted in significantly decreased expression of RFC genes in prostate cancer cells. The expression levels of *RFC* genes were determined by RNA sequencing. Log_2(Fold Change (siBTF3 versus siNC)) and adjusted *p* values are shown.

Supplementary Figure 5. Overexpression of BTF3a in prostate cancer cells does not affect the expression of RFC genes. a Western blot analysis of BTF3 protein abundance in DU145 and PC-3 cells with or without overexpression of BTF3a or C terminal V5-tagged BTF3b. Vinculin was used as a loading control. Note: in this experiment, the LI-COR Odyssey Imaging System was used for the near infrared fluorescent detection of the BTF3 antibody (rabbit host) and the V5 tag antibody (mouse host) simultaneously. **b** qRT-PCR analysis of the *RFCs* mRNA levels was conducted in DU145 and PC-3 cells with or without ectopic overexpression of BTF3a. *ACTB* was used as an endogenous control. Data are shown as mean ± S.D. for three independent experiments (n.s., not significant by student's *t*-test).

Supplementary Figure 6. The effect of BTF3 gene expression on cisplatin sensitivity in prostate cancer cells. a Cell viability assay of siRNA-mediated BTF3 knockdown in DU145 or control cells treated with or without cisplatin. **b** Cell viability assay of BTF3a-overexpressing DU145 or control cells treated with or without cisplatin. **c-d** The effect of BTF3b or BTF3a on the response of PC-3 cells to cisplatin. **e** The effect of RFC3 silencing on the response of BTF3b-overexpressing DU145 cells to

cisplatin. Cells were exposed to cisplatin for 2 hours and then subjected to fresh media for 3 days before MTT assay. Data are shown as mean ± S.D. for three independent experiments (* $p < 0.05$, ** $p < 0.01$; n.s., not significant by student’s *t*-test).
